# Therapeutic Use of Hygienic Measures

**Published:** 1850-03

**Authors:** John McLean

**Affiliations:** Prof. of Mat. Med. &c., in Rush Medical College


THE NORTH-WESTERN

MEDICAL AND SURGICAL JOURNAL.

VOL. II.] MARCH, 1850. [No. 6.

### Part I.—Original Communications.

### ARTICLE I.

*Therapeutic Use of Hygienic Measures.*—(Extracted from the Introductory Lecture of JOHN McLEAN, M.D., Prof. of Mat. Med. &c., in Rush Medical College: Session of 1849–50.)

Hygiene is intimately connected with Medical Science; and its nature is such, that we think it may be advantageously considered in connection with general therapeutics. It is a branch of medical edncation, in the principles of which the student of medicine should be thoroughly instructed.

By Hygiene is understood that science, which instructs us how to preserve health. Hygienic agents are such as air, light, aliment, exercise, clothing, excretions, sleep, and affections of the mind. In our course of lectures, we shall not only consider how they may be employed for the purpose of preserving health, but also, for the removal of diseased action.

By the ancients, these agents were called by the absurd name of non-naturals,—a much better appellation would have been naturals; for their proper use is absolutely essential to health, and also for the removal of disease, which, by their judicious management may many times be mitigated, or even cured, without the interference of medicine.

Hygiene has been too much neglected in medical education. It has been almost entirely overlooked, and the physician is left to acquire it, by a long course of observation and practice. Dr. Combe, in his principles of Physiology, says: "I have deplored the ignorance of the profession on this subject, which is the result of the little attention, that has been paid to it in the schools. The prominent aim of medicine, being to discriminate and cure disease, both the physician and the student fix upon that as their chief object, and are consequently apt to overlook the indirect but substantial aid, which an acquaintance with the laws of health is calculated to afford in restoring, as well as in preserving the healthy from disease. It is true, that almost every medical man, sooner or later, works out this knowledge for himself; but, in general, he attains it later than he ought to do; and seldom so completely as he would have done, had it been made a part of his elementary education, to which he saw others attaching importance."

"In my own instance, (says Dr. Combe,) it was only when entering upon practice, that I had first, occasion to feel and observe the evils arising from the ignorance which prevails in society in regard to it."

That physician who is acquainted with the principles of Hygiene, and acts accordingly, is much more successful in practice, all other things being equal, than he who is entirely ignorant on the subject. And why should we wonder at it; for when ignorant of the laws of health, the physician may prescribe his nauseous drugs for the purpose of combating disease, while at the same time, the hygienic condition may be such as to produce and foster the very disease he, by his remedies, is trying to overcome; and notwithstanding, they are thus working against his remedies, are entirely overlooked, and medicines are forced down in greater varieties and larger quantities, trying to effect that, which a little attention to the laws of health might speedily produce.

The more we see of disease and practice, the more are we convinced of the importance of Hygiene, and the necessity of its being made, more than it ever has been, a regular branch of medical education.

The student should study it systematically, with care and attention, until he becomes familiar with its principles; and it too, should be taught in all our schools of medicine.

The physician who has been long in practice, must have seen many diseases where the administration of mere drugs was attended with but very little benefit; but which, when accompanied with the proper hygienic treatment, were easily and speedily removed.

That physician who understands well the laws of health, and acts accordingly, and instructs others and his patients in the same principles, is truly a benefactor of mankind,—much more so than he, who is ignorant on this matter, disregards it entirely, and passes on through life, administering to the sick his medicines, regardless of those things, which may be more necessary to the welfare of his patients, than the medicines, upon which is placed so much dependence.

The practice of medicine has often been brought into disrepute, in consequence of not paying sufficient attention to this department. In many cases, medicines are given month after month and year after year, without any apparent benefit, in consequence of trusting altogether to their use. This and that physician are consulted. Medicines of various kinds are taken in great quantities, until the system has become completely drugged, and all, too, without any good resulting.—Now, tired of this, the system of infinitesimal doses is resorted to, which may amuse the imagination; and while it is productive of no positive injury, may, (in consequence of the suspension of an injurious medication,) be, apparently, productive of some good. But, in many instances, where the disease might be removed by a proper attention to the principles of Hygiene, in connection with a well directed course of medicine, it, too, fails, in consequence of neglecting those things, so necessary for the restoration of health.

Finally, in consequence of this non-success, the patient becomes disgusted with all systems and kinds of medicines, when, if he had only attended to a few rules of Hygiene, he might speedily have regained his health. Cases of this kind are many, and are almost daily presenting themselves to the practitioner. In order to illustrate the subject, we will suppose a case, and one too, that is of freqnent occurrence.—Here is an individual, who confines himself to his room, and there is fixed in an awkward and unnatural position, engaged, it may be, in writing, or some mechanical pursuit. He takes but little exercise, and gratifies his appetite with much, and many kinds of food, which is not the most easy of digestion.—He soon experiences a sense of illness, wandering pains, loss of appetite, a furred tongue, disordered digestion, constipated or relaxed bowels, lowness of spirits, and restless nights. A physician is consulted, and medicines are prescribed and taken; but the causes which produced the disease are still in full operation. For a few days, the medicine may appear to do good; but, in a little while, all the unpleasant symptoms return with increased energy. Encouraged by the slight relief that was obtained, he resorts to the same measures again, and with the same result.

In this manner, violating the laws of health, he is kept along month after month, and it may be, year after year, and is growing no better. Now, in such a case, the patient would be much more likely to regain his health, by acting in accordance with the principles of Hygiene, and taking less medicine.

In combating disease, we should lay hold of every means in our power, no matter whether they are dignified with the name of medicine or not. Whenever we can, we should call Hygiene to our aid; and for the restoration of health, it and medicine should work together.

Every physician who has been in practice for any length of time, must frequently have noticed, in many functional diseases, the therapeutic effects resulting from a properly directed course of Hygienic treatment. Under their influence, the mind is diverted from itself, and delighted and supported with pleasing thoughts. The lungs receive that amount and quality of air, which will enable them healthfully to perform their office; the body receives that amount of exercise which its condition demands---it is acted upon by the proper degree of warmth, supported with the necessary quantity and quality of food, and refreshed with the proper hours of undisturbed repose; and no wonder, that under the combined influence of all these causes, many functional diseases should give way to their health restoring power. When properly employed, they

> "Exalt each joy, allay each grief,
> Expel disease, and soften every pain."

How much of the good produced, in the treatment of disease upon the principles of Homœopathy, is due to the influence of these causes, we will not, now, pretend to say; but will leave it for you and others, who shall investigate the matter, to judge for yourselves.

If these agents are so beneficial, that of their opposites must be deleterious in the extreme. Were it necessary, we might go on, and mention a long catalogue of their evils, at the end of which steps in despair and snatches away the last remaining cup of human consolation.

> "Dire was the tosssing, deep the groans,
> Despair tended the sick, busiest from couch
> To couch; and over them triumphant death
> His dart shook, but delayed to strike,
> Tho' oft invoked with voice, as their chief
> Good and final hope."

In the light we have been viewing this subject, it directs us how to employ these agents as therapeutic means; and as such, the consideration of it, properly belongs to general therapeutics; and we consider, that a course of instruction on this subject, cannot be complete, nor what it should be, if this be neglected. The physician should understand how these agents should be used, so as best to preserve and restore health; and as it is closely connected with our department, we shall take the privilege of speaking upon the subject somewhat in detail, in our course of Lectures upon Materia Medica and general Therapeutics.

